# Supplementary material for: Single-cell transcriptomes reveal a molecular link between diabetic kidney and retinal lesions
Source: Commun Biol. 2023 Sep 5;6:912. doi: 10.1038/s42003-023-05300-4 (PMC10480496; doi:10.1038/s42003-023-05300-4)
Supplement: Supplementary file 2 — Supplementary Information [file 42003_2023_5300_MOESM2_ESM.pdf]

Supplementary materials for

Single-cell transcriptomes reveal a molecular link between diabetic kidney and retinal lesions

Ying Xu[1], Zhidan Xiang[1], Weigao E[2], Yue Lang[1], Sijia Huang[3], Weisong Qin [1], Jingping Yang[4, *], Zhaohong Chen[1, *], Zhihong Liu[1, *]

This PDF file includes:

1. **Two Supplementary Tables**

Supplementary Table. 1 Clinical characteristics of DN patients complicated without and with DR.

Supplementary Table. 2 Canonical marker genes used to annotation clusters.

2. **Five Supplementary Figures**

Supplementary Figure. 1 t-SNE plot of human kidney and retina.

Supplementary Figure. 2 Integration of human kidney and retina scRNA-seq data visualized through UMAP plots using Seurat 3.

Supplementary Figure. 3 Immunofluorescence staining of markers of HMCs and HRPCs.

Supplementary Figure. 4 The specifically expressed of CSPG4 in kidney.

Supplementary Figure. 5 Phenotype of DN and DR in *db/db* mice.

Supplementary Figure. 6 Calibration plots of observed and linear fitting for DN patients grouped by chemokine scores.

Supplementary Table.1 Clinical characteristics of DN patients complicated without and with DR.

	DN with DR patients (n = 14)	DN without DR patients (n = 15)	P value
Age (years)	46.373±8.299	47±8.058	0.8383
BMI (kg/m^2)	26.182±2.182	24.31±2.44	0.0381
HbA1c (%)	7.05±0.924	7.257±1.621	0.6732
eGFR(ml/min/1.73m^2)	75.447±30.304	58.015±26.867	0.1138
Proteinuria (g/24h)	2.152±3.422	4.385±3.456	0.0920
SBP (mmHg)	138.2±15.025	155.929±24.687	0.0261
DBP (mmHg)	81.6±10.986	89.429±11.673	0.0737

Supplementary Table.2 Canonical marker genes used to annotation clusters

Cell type	Markers
mesangial (MC)	*Pdgfrb*, *Cd34*, *Itga8*
glomerular capillary endothelial cells (GC-EC)	*Pecam1*, *Emcn*, *Flt1*
podocytes	*Nphs1*, *Nphs2*
B cells	*Cd79a*, *Cd79b*, *Ly6d*, *Mzb1*
T cells	*Bcl11b*, *Cxcr6*, *Fam189b*
neutrophils	*S100a9*, *S100a8*, *Retnlg*
macrophages	*Cybb*, *Coro1a*, *Pld4*
retinal pericytes (RPC)	*Pdgfrb*, *Myl9*, *Fhl2*
retinal endothelial cells (EC-R)	*Pecam1*, *Cdh5*, *Kdr*

Supplementary Figure. 1 t-SNE plot of human kidney and retina.

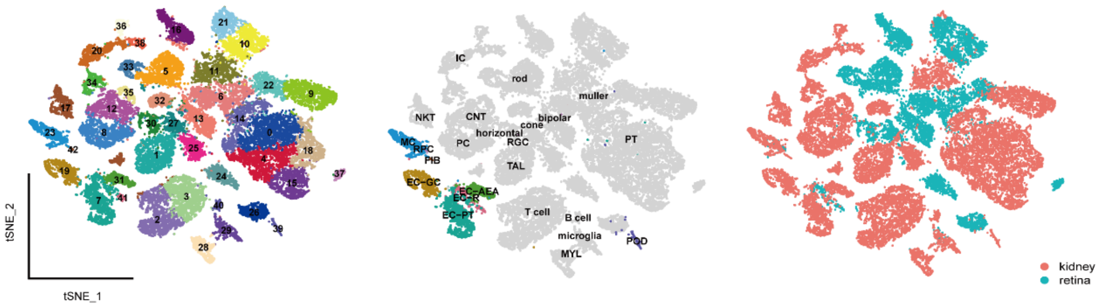

t-distributed stochastic neighbor embedding (t-SNE) showed that MCs and RPCs are grouped together in Cluster 23 and the sample types of origin are shown. Labeled numbers represent different cell clusters visualized by t-SNE. MC, mesangial cell, RPC, retinal pericyte, FIB, fibroblast, EC-GC, glomerular endothelial cell, EC-AEA, arteriolar endothelial cell, EC-PT, endothelial cell in peritubular, EC-R, retinal endothelial cell, POD, podocyte, CNT, connecting tubule, IC, intercalated cell, PC, principal cell, PT, proximal tubule cell, TAL, thick ascending limb cell, RGC, retinal ganglion cell, bipolar, retinal bipolar cell, cone, cone cell, horizontal, retinal horizontal cell, rod, rod cell, MYL, myeloid cell, NKT, natural killer T cell.

Supplementary Figure. 2 Integration of human kidney and retina scRNA-seq data visualized through UMAP plots using Seurat 3.

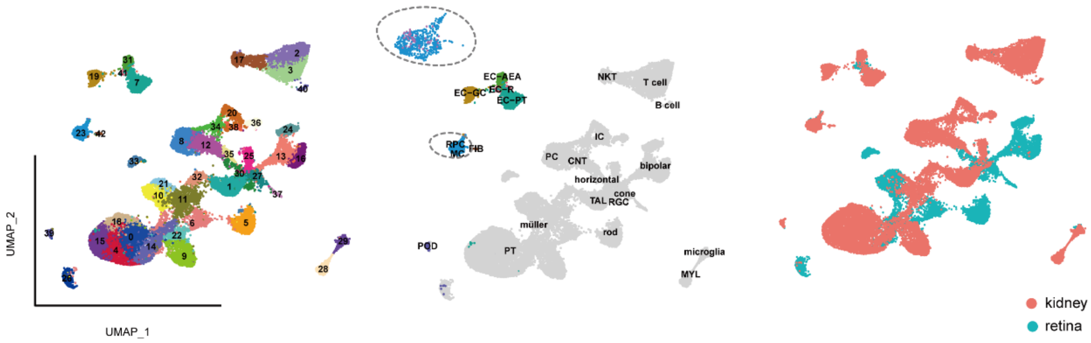

MCs and RPCs are grouped together by Seurat 3 and sample types of origin are shown.

Supplementary Figure. 3 Immunofluorescence staining of markers of HMCs and HRPCs.

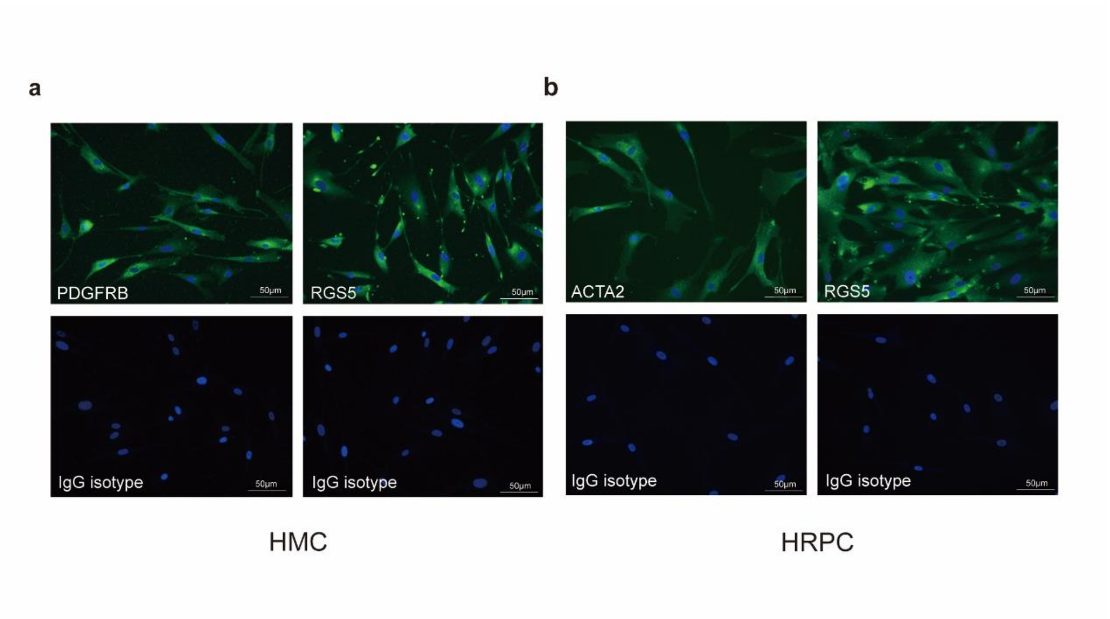

a. HMCs were stained with PDGFRB and RGS5, and **b.** HRPCs were stained with ACTA2 and RGS5, while the IgG isotypes were used as control. Scale bar: 50 μm.

Supplementary Figure. 4 The specific expression of CSPG4 in kidney.

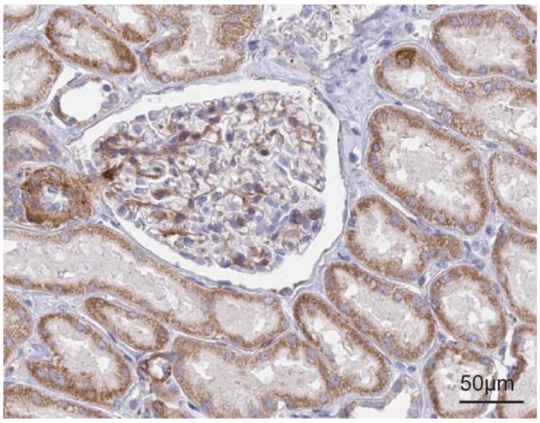

Data available in the Human Protein Atlas verified the specific expression of CSPG4 in human MCs. Scale bar: 50 μm.

Supplementary Figure. 5 Phenotype of DN and DR in *db/db* mice.

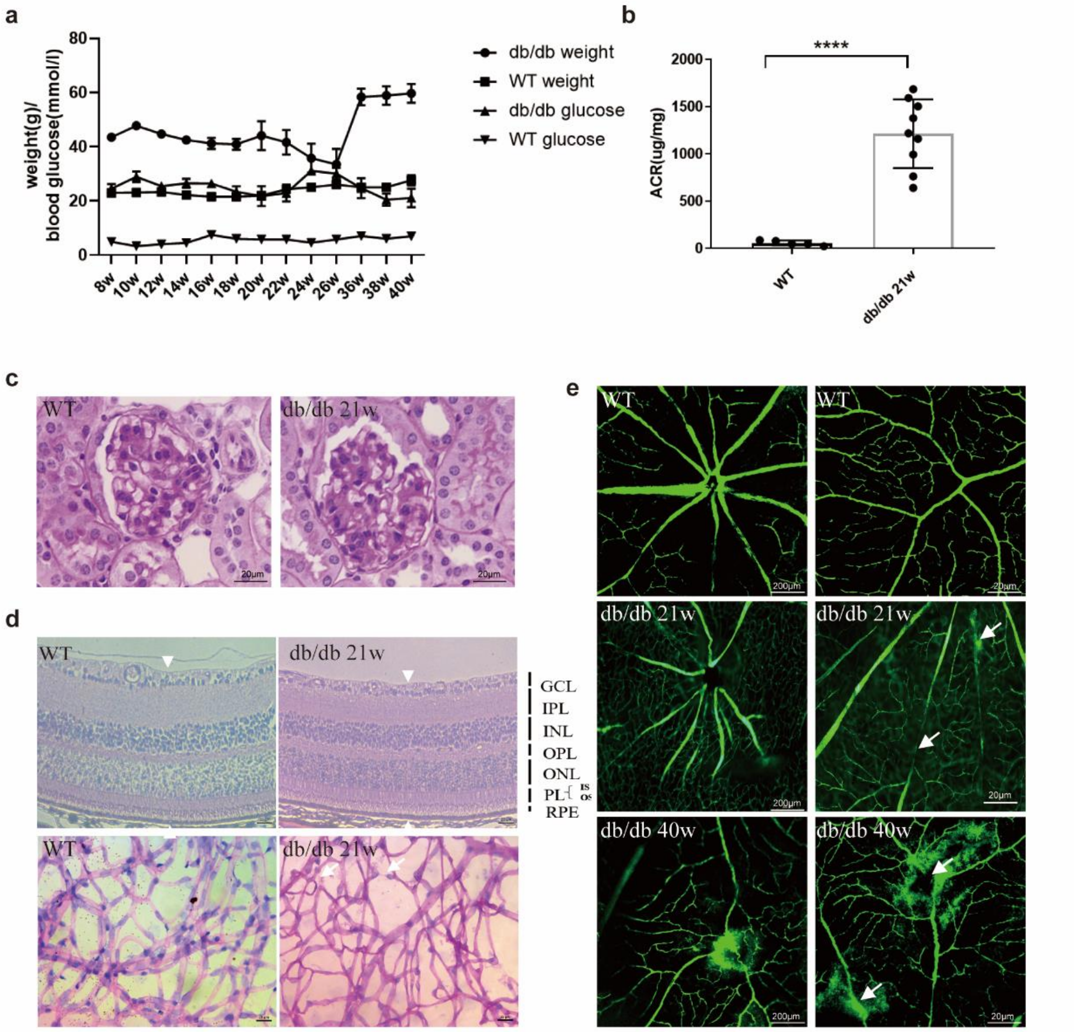

a. Body weight and fasting glucose of *db/db* mice were significantly higher than those of wt mice. **b.** The urinary albumin-creatinine ratio (ACR) of *db/db* mice was elevated at 21 weeks of age. **c.** The kidney sections of wt and *db/db* mice stained with PAS showed an increase in ECM in *db/db* mice. Scale bar: 20 µm. **d.** Retinal sections stained with H&E. The thickness of the retina became thinner and the number of acellular capillaries was increased in *db/db* mice. Scale bar: 20 µm. **e.** Fluorescence perfusion of mouse retinas at 21 and 40 weeks showed that retinal vessels became tortuous and

narrow, and capillary leakage occurred in *db/db* mice. Scale bar: 200 μm for main images and 20 μm for detail images. GCL, ganglion cell layer, IPL, inner plexiform layer, INL, inner nuclear layer, OPL, outer plexiform layer, ONL, outer nuclear layer, PL, plexiform layer, OS, outer segment; IS, inner segment, RPE, retinal pigmented epithelium.

Supplementary Figure. 6 Calibration plots of observed and linear fitting for DN patients grouped by chemokine scores.

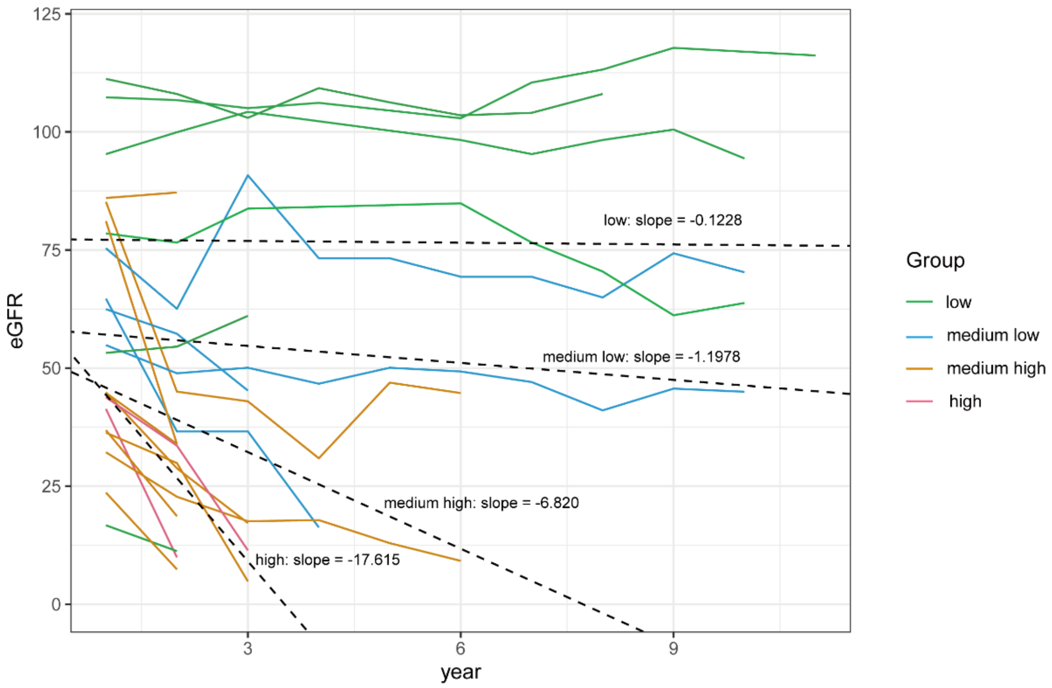

DN patients with high chemokine scores show rapid declines in eGFR.
