# Supplementary material for: Single-cell transcriptomes reveal a molecular link between diabetic kidney and retinal lesions
Source: Commun Biol. 2023 Sep 5;6:912. doi: 10.1038/s42003-023-05300-4 (PMC10480496; doi:10.1038/s42003-023-05300-4)
Supplement: Supplementary file 3 — Description of Additional Supplementary Files [file 42003_2023_5300_MOESM3_ESM.pdf]

Description of Additional Supplementary Files

File name: Supplementary Data 1.
Description: Gene expression in each cell cluster identified by Seurat in healthy human kidney and retina. Table indicating the top 20 representative genes of each cluster, identified by comparing the gene expression of each cluster against all others in the integrated dataset.

File name: Supplementary Data 2.
Description: Gene expression in each cell cluster identified by Seurat in glomeruli and retinas of wide type mice. Table indicating the top 20 representative genes of each cluster, identified by comparing the gene expression of each cluster against all others in the integrated dataset.

File name: Supplementary Data 3.
Description: Gene expression in each cell cluster identified by Seurat in glomeruli and retinas of *db/db* mice. Table indicating the top 20 representative genes of each cluster, identified by comparing the gene expression of each cluster against all others in the integrated dataset.

File name: Supplementary Data 4.
Description: Comparison of highly expressed genes in the normal control groups of HMC and HRPC. The top 2000 highly expressed genes of HMC and HRPC respectively and the overlap of them.

File name: Supplementary Data 5.
Description: Differential expression genes in HMC and HRPC induced by AGEs. Table indicating the up- and down-regulated genes in HMC and HRPC.

File name: Supplementary Data 6
Description: Source data for Figures 3c, 4d-f, and 6.
