# Supplementary material for: Single-cell transcriptomes reveal a molecular link between diabetic kidney and retinal lesions
Source: Commun Biol. 2023 Sep 5;6:912. doi: 10.1038/s42003-023-05300-4 (PMC10480496; doi:10.1038/s42003-023-05300-4)
Supplement: Supplementary file 10 — Reporting Summary [file 42003_2023_5300_MOESM10_ESM.pdf]

nature portfolio

Corresponding author(s): Zhihong Liu

Last updated by author(s): Aug 12, 2023

Reporting Summary

Nature Portfolio wishes to improve the reproducibility of the work that we publish. This form provides structure for consistency and transparency in reporting. For further information on Nature Portfolio policies, see our Editorial Policies and the Editorial Policy Checklist.

Statistics

For all statistical analyses, confirm that the following items are present in the figure legend, table legend, main text, or Methods section.

n/a	Confirmed	
☐	☒	The exact sample size (*n*) for each experimental group/condition, given as a discrete number and unit of measurement
☐	☒	A statement on whether measurements were taken from distinct samples or whether the same sample was measured repeatedly
☐	☒	The statistical test(s) used AND whether they are one- or two-sided<br>*Only common tests should be described solely by name; describe more complex techniques in the Methods section.*
☒	☐	A description of all covariates tested
☒	☐	A description of any assumptions or corrections, such as tests of normality and adjustment for multiple comparisons
☐	☒	A full description of the statistical parameters including central tendency (e.g. means) or other basic estimates (e.g. regression coefficient) AND variation (e.g. standard deviation) or associated estimates of uncertainty (e.g. confidence intervals)
☐	☒	For null hypothesis testing, the test statistic (e.g. *F*, *t*, *r*) with confidence intervals, effect sizes, degrees of freedom and *P* value noted<br>*Give P values as exact values whenever suitable.*
☒	☐	For Bayesian analysis, information on the choice of priors and Markov chain Monte Carlo settings
☒	☐	For hierarchical and complex designs, identification of the appropriate level for tests and full reporting of outcomes
☒	☐	Estimates of effect sizes (e.g. Cohen's *d*, Pearson's *r*), indicating how they were calculated

Our web collection on statistics for biologists contains articles on many of the points above.

Software and code

Policy information about availability of computer code

Data collection	We used the Cell Ranger (v3.0.0) from 10× Genomics to perform initial data demultiplexing, read alignment, UMI counting and annotation on the raw read data.
Data analysis	We used Seurat package (v3.2.3) for quality control filtering, data preprocessing, scaling, normalization, Louvain clustering and data visualization, UMAP algorithm for nonlinear dimensionality reduction. DEsingle (v1.14.0) for differential expressed genes calculation, clusterProfile (v3.14.3) for Gene Ontology analysis, MsigDB (v5.0) and Gene Set Variation Analysis package (v1.42.0) for ranked gene set enrichment analysis. Limma (v3.50.1) for differential expressed genes calculation in affymetrix transcriptional data, pROC (v1.18.0) for receiver operating characteristic curves. DESeq2 (v1.20.0) for differential expressed genes calculation in RNA-seq, ggplot2 (v3.0.0) and pheatmap (v1.0.12) for data plotting.

For manuscripts utilizing custom algorithms or software that are central to the research but not yet described in published literature, software must be made available to editors and reviewers. We strongly encourage code deposition in a community repository (e.g. GitHub). See the Nature Portfolio guidelines for submitting code & software for further information.

Data

Policy information about availability of data

All manuscripts must include a data availability statement. This statement should provide the following information, where applicable:

- Accession codes, unique identifiers, or web links for publicly available datasets
- A description of any restrictions on data availability
- For clinical datasets or third party data, please ensure that the statement adheres to our policy

Raw and processed scRNA-seq and RNA-seq data for this study have been deposited in the Gene Expression Omnibus database under accession number GSE204880 (secure token: ulotqemuvvmvxkr). Human kidney scRNA-seq data was downloaded from Rajasree et al. (2020) [GSE140989]. Human retina scRNA-seq data was downloaded from Andrew et al. (2020) [GSE142449]. Human glomruli Affymetrix microarray data was downloaded from Pan et al. (2017) [GSE96804].

Human research participants

Policy information about studies involving human research participants and Sex and Gender in Research.

Reporting on sex and gender	Reporting on sex and gender is not relevant to our study because we did not perform human research.
Population characteristics	Population characteristics is not relevant to our study because we did not perform human research.
Recruitment	Recruitment is not relevant to our study because we did not perform human research.
Ethics oversight	Ethics oversight is not relevant to our study because we did not perform human research.

Note that full information on the approval of the study protocol must also be provided in the manuscript.

Field-specific reporting

Please select the one below that is the best fit for your research. If you are not sure, read the appropriate sections before making your selection.

☒ Life sciences ☐ Behavioural & social sciences ☐ Ecological, evolutionary & environmental sciences

For a reference copy of the document with all sections, see nature.com/documents/nr-reporting-summary-flat.pdf

Life sciences study design

All studies must disclose on these points even when the disclosure is negative.

Sample size	For RNA-seq, 3 duplicates was common and adequate in studies. For scRNA-seq, the samples were combined and no replication was performed. For clinical data analysis, the sample size was determined by the previous study (Pubmed ID: 29242313).
Data exclusions	We only exclude cells in data filtering during anaysis. For clinical data analysis, the patients without fundus examination results were excluded.
Replication	Findings derived from scRNA-seq dataset were confirmed independently in mice and cultured cells and immumoflurescence staining.
Randomization	Randomization of human participants is not relevant to our study, because we did not allocate samples into different groups.
Blinding	Blinding is not relevant to our study because we did not require separate experimental groups.

Reporting for specific materials, systems and methods

We require information from authors about some types of materials, experimental systems and methods used in many studies. Here, indicate whether each material, system or method listed is relevant to your study. If you are not sure if a list item applies to your research, read the appropriate section before selecting a response.

Materials & experimental systems

n/a	Involved in the study	
☐	☒	Antibodies
☐	☒	Eukaryotic cell lines
☒	☐	Palaeontology and archaeology
☐	☒	Animals and other organisms
☒	☐	Clinical data
☒	☐	Dual use research of concern

Methods

n/a	Involved in the study	
☒	☐	ChIP-seq
☒	☐	Flow cytometry
☒	☐	MRI-based neuroimaging

Antibodies

Antibodies used

anti-mouse CD140b antibody (136006, BioLegend, 1:20); PE-anti platelet and endothelial cell adhesion molecule 1 antibody (160204, eBioscience, 1:20); anti-CSPG4 (sc-33666, Santa Cruz, 1:100); anti-PDGFRB antibody (ab32570, Abcam, 1:100); anti-αSMA antibody (ab32575, Abcam, 1:100); anti-NG2 antibody (ab183929, Abcam, 1:100); anti-CXCL1 antibody (12335-1-AP, proteintech, 1:100); FITC-conjugated rabbit anti-human IgG(H+L) (A0562, Beyotime Biotechnology, 1:500); Cy3-conjugated goat anti-rat IgG(H+L) (A0521, Beyotime Biotechnology, 1:500)

Validation

All antibodies are commercial available and were validated by the antibody manufacturer.
https://www.biolegend.com/en-us/products/pe-anti-mouse-cd140b-antibody-6256
https://www.biolegend.com/en-us/products/pe-anti-mouse-cd31-pecam-1-antibody-19418
https://www.scbt.com/p/ng2-antibody-132-38?requestFrom=search
https://www.abcam.cn/pdgfr-alpha--pdgfr-beta-antibody-y92-c-terminal-ab32570.html
https://www.abcam.cn/alpha-smooth-muscle-actin-acetyl-e3--actg2-acetyl-e3-antibody-e184-ab32575.html
https://www.abcam.cn/ng2-antibody-epr20244-ab183929.html
https://www.ptgcn.com/products/CXCL1-Antibody-12335-1-AP.htm
https://www.beyotime.com/product/A0562.html
https://www.beyotime.com/product/A0521.html

Eukaryotic cell lines

Policy information about cell lines and Sex and Gender in Research

Cell line source(s)

Primary Human Glomerular Mesangial Cells (ACBRI 127, cell systems)
Human Retinal Pericyte Cells (ACBRI 183, cell systems)

Authentication

Passed quality controls for characterization (by genotyping), viability and sterility by the manufacturer.

Mycoplasma contamination

Passed mycoplasma test by the manufacturer.

Commonly misidentified lines
(See ICLAC register)

No commonly misidentified cell lines were used in the study.

Animals and other research organisms

Policy information about studies involving animals; ARRIVE guidelines recommended for reporting animal research, and Sex and Gender in Research

Laboratory animals

21-week old wide type and db/db in C57BLKs/J background mice. Mice were purchased from the Model Animal Research Center of Nanjing University and maintained in constant-temperature rooms and humidity under a 12-hour light/dark cycle.

Wild animals

No wild animals were used in the study.

Reporting on sex

Only male mice were used in the study.

Field-collected samples

No field collected samples were used in the study.

Ethics oversight

Animal experiments have been approved by the Institutional Animal Care and Use Committee of Jinling Hospital.

Note that full information on the approval of the study protocol must also be provided in the manuscript.
